# Supplementary material for: Pyruvate Dehydrogenase Kinase 1 inhibition mediated oxidative phosphorylation enhancement in cartilage promotes osteoarthritis progression
Source: BMC Musculoskelet Disord. 2023 Jul 20;24:597. doi: 10.1186/s12891-023-06585-6 (PMC10357736; doi:10.1186/s12891-023-06585-6)
Supplement: Supplementary file 1 — Additional file 1. [file 12891_2023_6585_MOESM1_ESM.zip › MF.Gsea.1653627316242/GOMF_INWARD_RECTIFIER_POTASSIUM_CHANNEL_ACTIVITY.html]

Details for gene set GOMF\_INWARD\_RECTIFIER\_POTASSIUM\_CHANNEL\_ACTIVITY[GSEA]

|  || Dataset | 55457\_collapsed\_to\_symbols.cls.cls #OA\_versus\_normal.cls.cls #OA\_versus\_normal\_repos |
| Phenotype | cls.cls#OA\_versus\_normal\_repos |
| Upregulated in class | normal |
| GeneSet | GOMF\_INWARD\_RECTIFIER\_POTASSIUM\_CHANNEL\_ACTIVITY |
| Enrichment Score (ES) | -0.43868986 |
| Normalized Enrichment Score (NES) | -1.4095155 |
| Nominal p-value | 0.0723104 |
| FDR q-value | 0.26459607 |
| FWER p-Value | 1.0 |
Table: GSEA Results Summary

  

Fig 1: Enrichment plot: GOMF\_INWARD\_RECTIFIER\_POTASSIUM\_CHANNEL\_ACTIVITY      
 Profile of the Running ES Score & Positions of GeneSet Members on the Rank Ordered List

  

| SYMBOL | TITLE | RANK IN GENE LIST | RANK METRIC SCORE | RUNNING ES | CORE ENRICHMENT || 1 | ABCC8 | ATP binding cassette subfamily C member 8 [Source:HGNC Symbol;Acc:HGNC:59] | 2844 | 0.266 | -0.1750 | No |
| 2 | KCNH6 | potassium voltage-gated channel subfamily H member 6 [Source:HGNC Symbol;Acc:HGNC:18862] | 3066 | 0.243 | -0.1502 | No |
| 3 | KCNJ6 | potassium inwardly rectifying channel subfamily J member 6 [Source:HGNC Symbol;Acc:HGNC:6267] | 3551 | 0.198 | -0.1537 | No |
| 4 | KCNJ3 | potassium inwardly rectifying channel subfamily J member 3 [Source:HGNC Symbol;Acc:HGNC:6264] | 4072 | 0.149 | -0.1684 | No |
| 5 | KCNK1 | potassium two pore domain channel subfamily K member 1 [Source:HGNC Symbol;Acc:HGNC:6272] | 4235 | 0.134 | -0.1579 | No |
| 6 | KCNJ16 | potassium inwardly rectifying channel subfamily J member 16 [Source:HGNC Symbol;Acc:HGNC:6262] | 4693 | 0.097 | -0.1767 | No |
| 7 | KCNJ14 | potassium inwardly rectifying channel subfamily J member 14 [Source:HGNC Symbol;Acc:HGNC:6260] | 5096 | 0.065 | -0.1967 | No |
| 8 | KCNE2 | potassium voltage-gated channel subfamily E regulatory subunit 2 [Source:HGNC Symbol;Acc:HGNC:6242] | 5226 | 0.054 | -0.1974 | No |
| 9 | KCNJ2 | potassium inwardly rectifying channel subfamily J member 2 [Source:HGNC Symbol;Acc:HGNC:6263] | 6192 | -0.020 | -0.2689 | No |
| 10 | KCNJ5 | potassium inwardly rectifying channel subfamily J member 5 [Source:HGNC Symbol;Acc:HGNC:6266] | 6982 | -0.078 | -0.3167 | No |
| 11 | KCNH2 | potassium voltage-gated channel subfamily H member 2 [Source:HGNC Symbol;Acc:HGNC:6251] | 7724 | -0.136 | -0.3508 | No |
| 12 | KCNJ9 | potassium inwardly rectifying channel subfamily J member 9 [Source:HGNC Symbol;Acc:HGNC:6270] | 8857 | -0.222 | -0.4005 | Yes |
| 13 | KCNJ15 | potassium inwardly rectifying channel subfamily J member 15 [Source:HGNC Symbol;Acc:HGNC:6261] | 9025 | -0.234 | -0.3731 | Yes |
| 14 | KCNN1 | potassium calcium-activated channel subfamily N member 1 [Source:HGNC Symbol;Acc:HGNC:6290] | 9289 | -0.255 | -0.3496 | Yes |
| 15 | ABCC9 | ATP binding cassette subfamily C member 9 [Source:HGNC Symbol;Acc:HGNC:60] | 9346 | -0.259 | -0.3093 | Yes |
| 16 | KCNJ8 | potassium inwardly rectifying channel subfamily J member 8 [Source:HGNC Symbol;Acc:HGNC:6269] | 9421 | -0.266 | -0.2691 | Yes |
| 17 | KCNJ4 | potassium inwardly rectifying channel subfamily J member 4 [Source:HGNC Symbol;Acc:HGNC:6265] | 10080 | -0.329 | -0.2636 | Yes |
| 18 | KCNJ10 | potassium inwardly rectifying channel subfamily J member 10 [Source:HGNC Symbol;Acc:HGNC:6256] | 10666 | -0.390 | -0.2418 | Yes |
| 19 | KCNN3 | potassium calcium-activated channel subfamily N member 3 [Source:HGNC Symbol;Acc:HGNC:6292] | 10761 | -0.400 | -0.1802 | Yes |
| 20 | KCNJ12 | potassium inwardly rectifying channel subfamily J member 12 [Source:HGNC Symbol;Acc:HGNC:6258] | 11079 | -0.441 | -0.1287 | Yes |
| 21 | KCNJ13 | potassium inwardly rectifying channel subfamily J member 13 [Source:HGNC Symbol;Acc:HGNC:6259] | 11297 | -0.470 | -0.0644 | Yes |
| 22 | KCNJ1 | potassium inwardly rectifying channel subfamily J member 1 [Source:HGNC Symbol;Acc:HGNC:6255] | 11418 | -0.489 | 0.0106 | Yes |
| 23 | KCNN2 | potassium calcium-activated channel subfamily N member 2 [Source:HGNC Symbol;Acc:HGNC:6291] | 12048 | -0.605 | 0.0662 | Yes |
Table: GSEA details [plain text format]

  

Fig 2: GOMF\_INWARD\_RECTIFIER\_POTASSIUM\_CHANNEL\_ACTIVITY      
 Blue-Pink O' Gram in the Space of the Analyzed GeneSet

  

Fig 3: GOMF\_INWARD\_RECTIFIER\_POTASSIUM\_CHANNEL\_ACTIVITY: Random ES distribution      
 Gene set null distribution of ES for **GOMF\_INWARD\_RECTIFIER\_POTASSIUM\_CHANNEL\_ACTIVITY**
